# The Correlation Between Migraine Frequency and Sleep Disturbances in Adults: A Cross-Sectional Study

**DOI:** 10.7759/cureus.87282

**Published:** 2025-07-04

**Authors:** Wardah Ikram

**Affiliations:** 1 Emergency Medicine, Allama Iqbal Medical College, Lahore, PAK

**Keywords:** cross-sectional study, migraine, migraine frequency, pittsburgh sleep quality index, sleep disturbances

## Abstract

Introduction

Sleep disturbances and migraines are prevalent neurological conditions that can significantly impair an individual’s quality of life. Research suggests a bidirectional relationship, where migraines contribute to sleep disruption, and poor sleep quality may intensify migraine frequency and severity. This study aimed to assess the relationship between migraine frequency and sleep quality in adult patients. Additionally, it endeavored to explore the effects of migraine intensity and duration, gender differences, and identify independent predictors of poor sleep using logistic regression.

Methodology

A cross-sectional study was conducted over one year (January-December 2020) at the Department of Neurology, Allama Iqbal Medical College, Lahore. Using convenience sampling, 103 adult migraine patients were enrolled. The Pittsburgh Sleep Quality Index (PSQI) was used to assess sleep quality. Data on migraine frequency, duration, and intensity were collected. Statistical analyses included descriptive statistics, one-way analysis of variance (ANOVA), Pearson correlation, chi-square tests, independent t-tests, and binary logistic regression to identify predictors of poor sleep quality.

Results

The mean age of participants was 34.7 ± 10.8 years, with 69 (67%) females. Poor sleep quality (PSQI >5) was observed in 68 participants (66%). Mean PSQI scores increased significantly with migraine frequency (low: 5.8 ± 2.7; moderate: 8.1 ± 3.1; high: 10.5 ± 3.2; p<0.001). Pearson correlation analysis revealed a moderate positive association between migraine frequency and PSQI score (r = 0.54, p<0.001), indicating that as the number of migraine attacks per month increased, sleep quality worsened proportionally. A correlation coefficient (r) of 0.54 suggests a statistically meaningful and clinically relevant relationship, where migraine frequency accounts for a moderate proportion of the variability in sleep disturbance severity. This finding supports the hypothesis that migraine frequency plays a direct role in sleep impairment, warranting integrated clinical management strategies. Female participants reported higher migraine frequency and poorer sleep than males (p = 0.04). The prevalence of poor sleep rose from 18 participants (42.9%) in the low-frequency group to 24 (100%) in the high-frequency group (p<0.001). Logistic regression analysis revealed that female gender [odds ratio (OR) = 2.43, p = 0.04] and increased migraine frequency (OR = 1.46 per additional attack/month, p<0.001) were significant independent predictors of poor sleep quality.

Conclusions

Migraine frequency is strongly associated with poor sleep quality, with individuals experiencing more frequent attacks reporting significantly worse sleep. Migraine severity and duration also showed positive associations with impaired sleep, while female gender emerged as an independent predictor. These findings underscore the need to routinely assess and manage sleep quality in migraine patients, particularly among women and those with high-frequency attacks. Interventions aimed at improving sleep hygiene and treating comorbid sleep disturbances may serve as an important component of comprehensive migraine management. Future research should explore the effectiveness of targeted sleep interventions in reducing migraine burden.

## Introduction

Frequent episodes of moderate to severe headaches, often accompanied by nausea, photophobia, and phonophobia, are the hallmark of migraine, a common and debilitating neurological disorder [1]. Globally, approximately 12-15% of the population is affected, with a higher prevalence observed among women and individuals in their most productive years [2]. The burden of migraine is multifaceted, affecting physical health, emotional well-being, occupational performance, and overall quality of life [3]. Despite its prevalence, the pathophysiology of migraine remains complex, involving genetic, environmental, and neurovascular factors [4]. Recent studies have further highlighted the interplay between sleep and migraine: one investigation among university students found that 64% of migraineurs had poor sleep quality, significantly associated with aura symptoms, anxiety, and mobile phone addiction, especially in females [3]. Another study among young adult women revealed that migraine frequency, intensity, and disability were closely linked to specific sleep disruptions, such as daytime somnolence, reduced sleep adequacy, and snoring, reinforcing the role of sleep alterations in the migraine experience [4].

Among the many contributors to migraine attacks, sleep disturbances have emerged as significant and potentially modifiable factors [5]. Sleep is essential for neurological homeostasis, and disruptions such as insomnia, poor sleep quality, hypersomnia, and circadian rhythm disorders are frequently reported in patients with migraine [6]. Several neurobiological pathways, including those involving serotonin and melatonin regulation, may underlie this bidirectional relationship, wherein poor sleep can trigger migraine episodes, and migraines, in turn, can impair sleep quality [7]. Multiple studies have indicated that migraineurs report more frequent sleep disturbances compared to the general population [8-11]. These disturbances not only increase the frequency and severity of migraine attacks but may also reduce the effectiveness of treatment strategies. A cross-sectional study among Lebanese university medical students reported a 12.1% prevalence of migraine, with significant associations identified between migraine and factors such as anxiety, depression, insomnia, and online education. Over half of the students with migraine experienced severe disability as measured by the MIDAS scale, underscoring its substantial impact on daily functioning [12].

A study from eastern India demonstrated a moderate positive correlation between headache frequency and poor sleep quality in migraine patients, with chronic migraine sufferers showing significantly worse sleep parameters than those with episodic migraine (r = 0.45, p<0.01) [10]. Non-headache symptoms such as allodynia, nasal congestion, and cervical muscle tenderness were more prevalent in the chronic migraine group, contributing to greater overall disability [13]. Furthermore, specific populations (particularly in low- and middle-income countries) remain understudied due to limited awareness and diagnostic challenges, making it difficult to generalize existing findings [14].

Despite growing evidence, the association between migraine frequency and distinct patterns of sleep disturbances is still underexplored in adult populations, particularly within diverse sociocultural contexts. Therefore, this study aimed to assess the association between migraine frequency and sleep disturbances among adults by using a cross-sectional design. Additionally, it explored the effects of migraine intensity and duration, gender differences, and identified independent predictors of poor sleep by using logistic regression.

## Materials and methods

Study design and setting

This cross-sectional study was conducted over 12 months, from January to December 2020, at the Department of Neurology, Jinnah Hospital, Lahore, affiliated with Allama Iqbal Medical College. The estimated annual outpatient volume of the clinic is approximately 700,000. Participants were recruited through convenience sampling from the neurology outpatient department. Adults aged 18-65 years with a clinical diagnosis of episodic or chronic migraine were eligible for inclusion. Patients with comorbid neurological or psychiatric conditions or a history of substance abuse were excluded. Only individuals who were willing to participate after being briefed about the study objectives and procedures were included. 

Inclusion and exclusion criteria

The study included adults aged 18-60 years with a clinical diagnosis of migraine according to the International Classification of Headache Disorders, 3rd edition (ICHD-3) criteria [15]. Patients with comorbid neurological disorders, psychiatric disorders other than sleep disorders, or chronic medical conditions known to influence sleep (such as obstructive sleep apnea, central sleep apnea, or restless leg syndrome) were excluded. While formal sleep studies (e.g., polysomnography) were not conducted, all participants were evaluated for sleep-related disorders through detailed clinical history and physician examination. Patients with clinical suspicion of central or obstructive sleep apnea were excluded.

To screen for psychiatric comorbidities, a structured clinical interview was conducted by a trained physician based on the Diagnostic and Statistical Manual of Mental Disorders, Fifth Edition (DSM-5) criteria [16]. The Patient Health Questionnaire-9 (PHQ-9) was used to assess depressive symptoms, with a score of ≥10 indicating moderate to severe depression and leading to exclusion [17]. While anxiety was not assessed using a formal scale such as the General Anxiety Disorder-7 (GAD-7), clinical signs of anxiety were evaluated during the physician interview, and patients with clinically significant anxiety disorders were excluded.

Sampling technique and sample size

A total of 103 participants were recruited via non-probability consecutive sampling. This method was chosen due to its practical feasibility in a clinical setting, allowing for the inclusion of all eligible patients presenting during the study period. The sample size was calculated using OpenEpi version 3.01, assuming a confidence level (CI) of 95%, a margin of error of 10%, and an anticipated correlation coefficient (r) of 0.3 based on prior literature indicating a moderate correlation between migraine frequency and sleep disturbances [18]. The minimum required sample size was 91. To account for potential non-responses or incomplete datasets, the final sample size was increased to 103 participants.

Data collection

Demographic information, migraine clinical parameters (frequency, duration, and related symptoms), and sleep-related data were gathered using a standardized questionnaire. Per migraine categorization frameworks like the ICHD-3, migraine frequency was classified as mild (one to three attacks/month), moderate (four to seven attacks/month), and high (≥8 attacks/month) using criteria often used in clinical research. The Pittsburgh Sleep Quality Index (PSQI), a validated instrument for evaluating sleep quality and detecting sleep-related issues, was used to measure sleep disruptions. Poor sleep quality was thought to be indicated by a PSQI global score of more than 5 [19].

Data analysis

Data analysis was conducted using SPSS Statistics version 25 (IBM Corp., Armonk, NY). Clinical and demographic characteristics were summarized using descriptive statistics. Categorical variables, including gender, migraine frequency categories, and the presence of sleep disturbances, were presented as frequencies and percentages. Continuous variables such as age, migraine duration, and PSQI scores were expressed as mean ± standard deviation (SD). The Shapiro-Wilk test was applied to assess the normality of continuous data. The association between migraine frequency and PSQI scores was examined using Pearson’s correlation coefficient, with the strength and direction of the relationship interpreted based on standard benchmarks (small: r = 0.1-0.29, moderate: r = 0.3-0.49, strong: r≥0.5). The visual analog scale (VAS) was used to measure the intensity of migraines, and the results were connected with the quality of sleep. To assess variations in PSQI ratings and migraine frequency between male and female subjects, gender-based subgroup analyses were also conducted.

To compare sleep quality across different levels of migraine frequency (low, moderate, high), a one-way analysis of variance (ANOVA) was performed, followed by a post hoc Tukey test to identify significant pairwise differences. Independent samples t-tests were used to compare continuous variables, such as PSQI scores and migraine duration, between male and female participants. A chi-square (χ²) test was employed to evaluate the association between categorical variables, particularly the prevalence of poor sleep quality across migraine frequency groups. Binary logistic regression was used to determine independent predictors of poor sleep quality (PSQI >5). Covariates included gender, age, migraine frequency, migraine duration, PHQ-9 score, caffeine intake, and use of analgesic or sedative medications. These variables were selected based on prior literature to control for potential confounding effects. All statistical tests were two-tailed, and a p-value of less than 0.05 was considered statistically significant, ensuring a rigorous and comprehensive evaluation of the relationship between migraine characteristics and sleep quality.

Ethical considerations

Before data collection, the Institutional Review Board of Allama Iqbal Medical College, Lahore, issued approval (957/DN/AIMC). This study was carried out in compliance with the hospital's institutional research committee's ethical guidelines. Written informed consent was obtained from all participants before enrollment.

## Results

**Figure 1:**
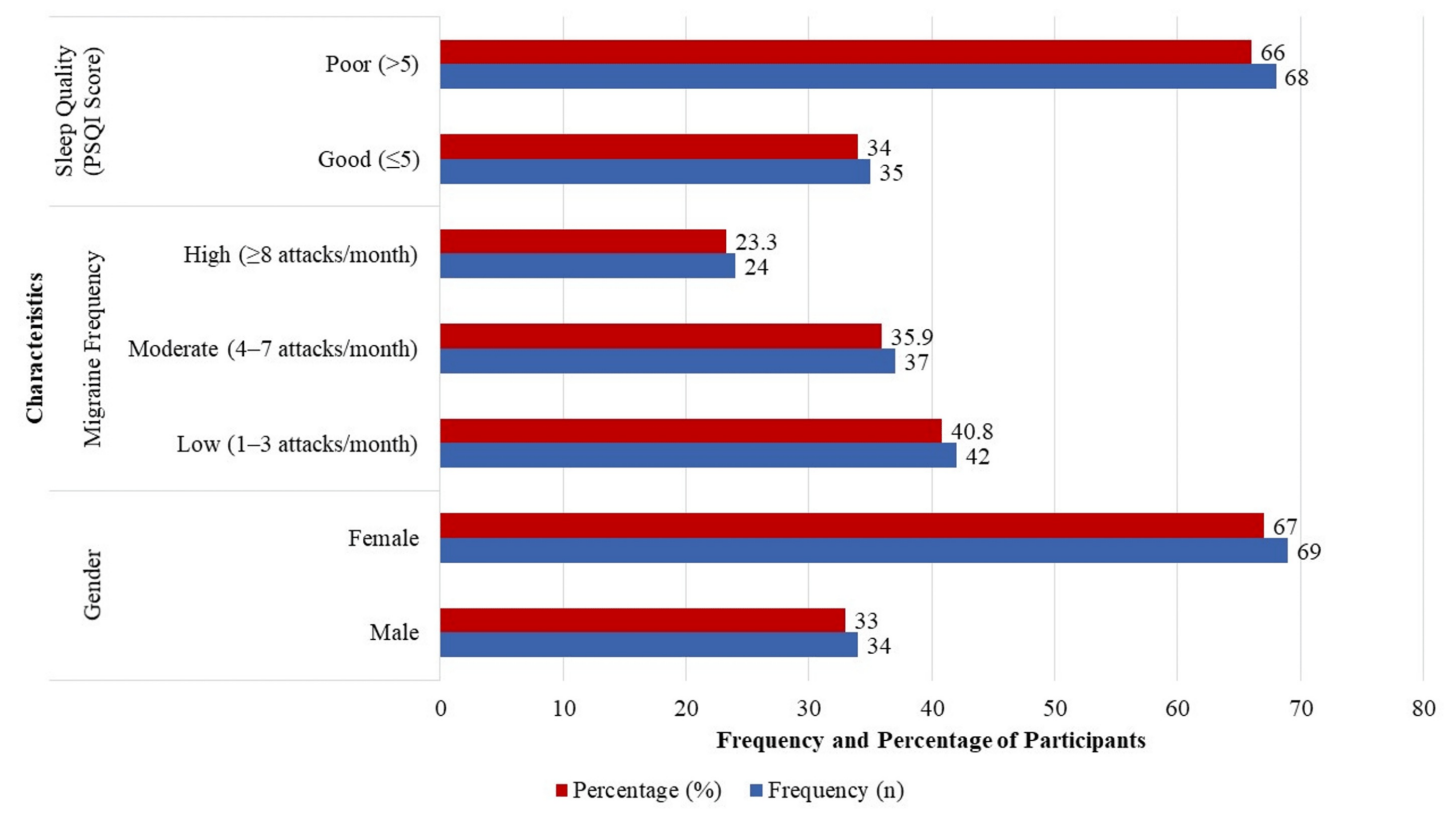
Demographic and clinical characteristics (n=103) PSQI: Pittsburgh Sleep Quality Index

A total of 103 adult migraine patients participated in the study, with a mean age of 34.7 ± 10.8 years (range: 18-60 years). Females made up 67.0% (n = 69) of the sample, while males accounted for 33.0% (n = 34). Regarding migraine frequency, 42 participants (40.8%) experienced low frequency (one to three attacks per month), 37 (35.9%) had moderate frequency (four to seven attacks per month), and 24 (23.3%) reported high frequency (eight or more attacks per month). Participants' sleep quality was evaluated using PSQI. Of them, 68 (66.0%) had poor sleep quality (PSQI score >5), whereas 35 (34.0%) had excellent sleep quality (PSQI score <5). Figure 1 provides a summary of these clinical and demographic characteristics.

The overall mean PSQI score for the study sample was 7.9 ± 3.4, indicating a general trend toward poor sleep quality among participants. When analyzed by migraine frequency, mean PSQI scores showed a clear increase with the number of migraine attacks per month. Participants in the low-frequency group (one to three attacks/month) had a mean PSQI score of 5.8 ± 2.7, those in the moderate-frequency group (four to seven attacks/month) scored 8.1 ± 3.1, and the high-frequency group (≥8 attacks/month) reported the highest mean score of 10.5 ± 3.2. Statistical analysis using one-way ANOVA revealed a significant difference in sleep quality among the three groups [F (2,100) = 22.7, p<0.001]. Further post hoc analysis with Tukey’s test confirmed that the mean PSQI scores differed significantly between each pair of migraine frequency groups (p<0.05). These findings, summarized in Table 1, suggest that increasing migraine frequency is associated with progressively poorer sleep quality.

**Table 1:** Mean PSQI scores by migraine frequency (one-way ANOVA) ANOVA: analysis of variance; PSQI: Pittsburgh Sleep Quality Index; SD: standard deviation

Migraine frequency	PSQI, mean ± SD	F-value	P-value
Low frequency (n = 42)	5.8 ± 2.7	22.7	<0.001
Moderate frequency (n = 37)	8.1 ± 3.1		
High frequency (n = 24)	10.5 ± 3.2		

Patients with more frequent migraine episodes tended to have worse sleep quality, according to a moderately positive association between migraine frequency and PSQI score (r = 0.54, p<0.001) per Pearson correlation analysis. Additionally, there was a small but statistically significant positive link between the length of migraine history and PSQI scores (r = 0.29, p = 0.003), indicating that individuals who have had migraines for a longer period may have greater sleep problems. There was a moderate correlation between poor sleep quality and migraine severity as judged by VAS (r = 0.43, p<0.001). Age did not significantly affect PSQI ratings (r = -0.12, p = 0.22), suggesting that age did not have a major impact on migraine patients' sleep quality in this group (Table 2).

**Table 2:** Correlation between key variables and PSQI score (sleep quality) PSQI: Pittsburgh Sleep Quality Index; VAS: visual analog scale

Variables	Correlation coefficient (r)	P-value
Migraine frequency (attacks/month)	0.54	<0.001
Age (years)	-0.12	0.22
Duration of migraine history (years)	0.29	0.003
Migraine intensity (VAS score)	0.43	<0.001

The mean PSQI score for females (8.3 ± 3.5) was significantly higher than that of males (7.1 ± 3.0), indicating poorer sleep quality in female migraineurs (t(101) = 2.08, p = 0.04). Females also reported a higher mean migraine frequency (6.4 ± 3.3 attacks/month) compared to males (4.9 ± 2.7 attacks/month), which may partly explain their increased sleep disturbances. When migraine frequency categories were stratified by gender, PSQI scores increased consistently with migraine frequency in both males and females. Males with high-frequency migraines had a mean PSQI score of 10.3 ± 2.7, while females in the same category had a slightly higher score of 10.7 ± 3.4. The distribution of migraine frequencies was skewed toward higher frequencies among females, and females in each category tended to report slightly worse sleep quality (Table 3).

**Table 3:** Gender-wise distribution of PSQI scores, migraine frequency, and sleep quality PSQI: Pittsburgh Sleep Quality Index; SD: standard deviation

Group	PSQI, mean ± SD	Migraine frequency (attacks/month), mean ± SD	Poor sleep (PSQI >5)	Good sleep (PSQI ≤5)	Total (n)	% with poor sleep
Male (n = 34)	7.1 ± 3.0	4.9 ± 2.7	19	15	34	0.559
Male – low frequency (n = 18)	5.6 ± 2.2	2.3 ± 0.7	-	-	-	-
Male – moderate frequency (n = 10)	7.8 ± 2.9	5.5 ± 0.6	-	-	-	-
Male – high frequency (n = 6)	10.3 ± 2.7	9.2 ± 1.1	-	-	-	-
Female (n = 69)	8.3 ± 3.5	6.4 ± 3.3	49	20	69	0.71
Female – low frequency (n = 24)	5.9 ± 3.0	2.7 ± 0.8	-	-	-	-
Female – moderate frequency (n = 27)	8.2 ± 3.1	6.0 ± 0.7	-	-	-	-
Female – high frequency (n = 18)	10.7 ± 3.4	9.5 ± 1.0	-	-	-	-

**Figure 2:**
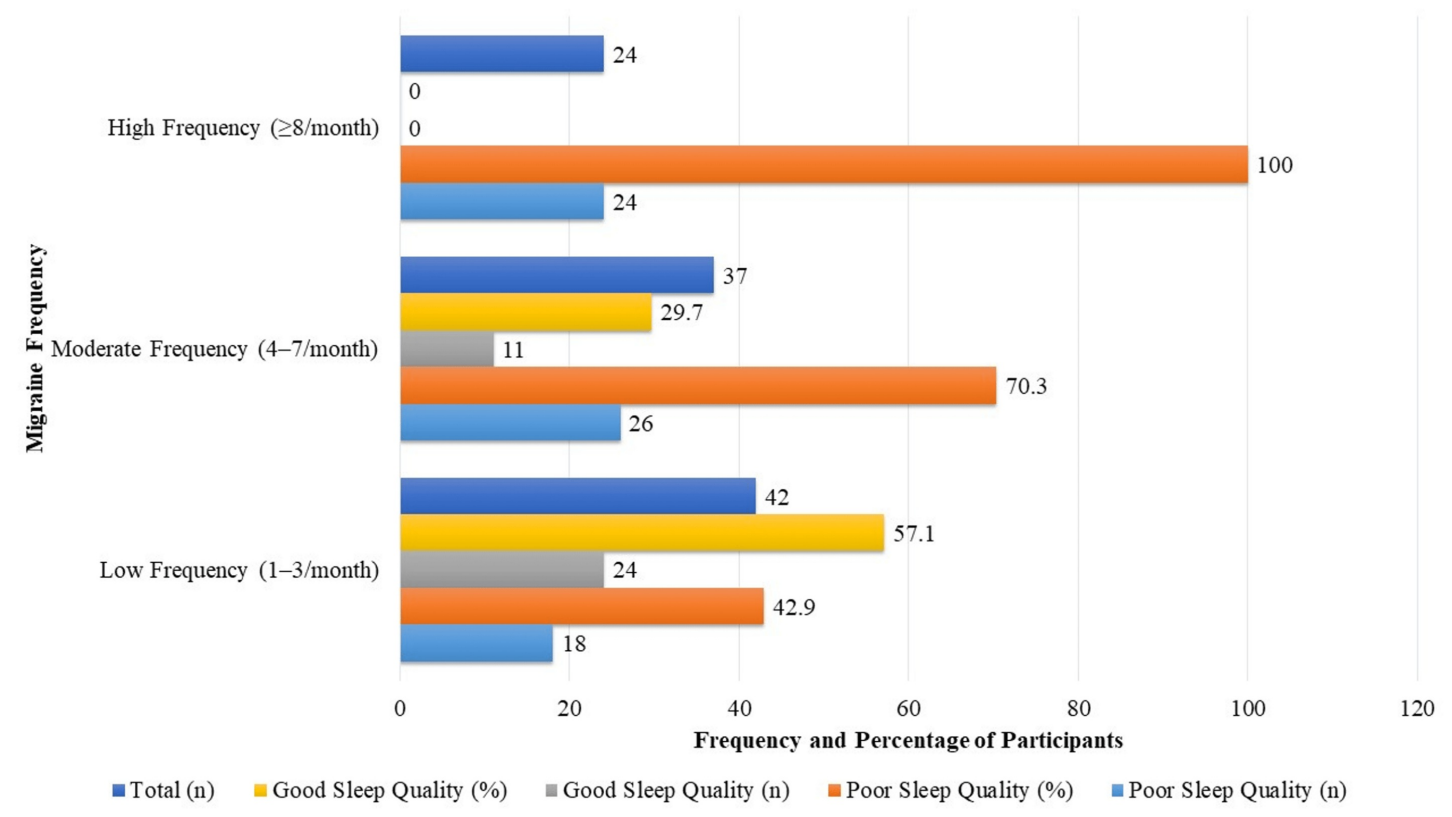
Prevalence of poor and good sleep quality by migraine frequency Chi-square analysis revealed a significant association between migraine frequency and sleep quality (χ² (2) = 26.4, p<0.001)

The prevalence of poor sleep quality (defined as a PSQI score >5) showed a marked increase with rising migraine frequency among participants. In the low-frequency group (one to three attacks/month), 18 out of 42 individuals (42.9%) reported poor sleep quality, while in the moderate-frequency group (four to seven attacks/month), this proportion rose to 26 out of 37 participants (70.3%). Strikingly, all 24 participants (100%) in the high-frequency group (≥8 attacks/month) experienced poor sleep quality. Conversely, good sleep quality (PSQI ≤5) was most common in the low-frequency group (24/42, 57.1%) and declined with increasing migraine frequency. A chi-square test demonstrated a statistically significant association between migraine frequency and prevalence of poor sleep quality (χ² (2) = 26.4, p<0.001), underscoring the relationship between more frequent migraine attacks and deteriorating sleep quality. These findings are illustrated in Figure 2.

Table 4 shows the findings of a comparison between male and female migraine patients on several variables related to sleep quality and migraine frequency. The PSQI is used to measure sleep quality, with scores >5 indicating poor sleep. Independent samples t-tests were used to compare continuous variables (PSQI score and migraine frequency), while the χ² test was applied to categorical variables (proportion of poor vs. good sleepers). Although females reported higher mean PSQI scores than males, the difference was not statistically significant (p = 0.085). However, females had a significantly higher mean migraine frequency than males (p = 0.029). The difference in the proportion of poor sleepers between genders was not statistically significant (p = 0.128), suggesting that gender alone may not account for variations in sleep quality.

**Table 4:** Gender differences in sleep quality and migraine frequency Independent samples t-tests were used for continuous variables; the chi-square test was used for the categorical association between gender and sleep quality; p-values <0.05 were significant. Although the difference in PSQI scores between genders was not statistically significant in univariate analysis (p = 0.085), logistic regression in Table 5 identified female gender as an independent predictor of poor sleep quality after adjusting for migraine frequency PSQI: Pittsburgh Sleep Quality Index; SD: standard deviation

Variable	Male (n = 34)	Female (n = 69)	Statistical test	P-value
PSQI score, mean ± SD	7.1 ± 3.0	8.3 ± 3.5	t = 1.74	0.085
Migraine frequency, mean ± SD	4.9 ± 2.7	6.4 ± 3.3	t = 2.21	0.029
Poor sleep quality (PSQI >5)	19 (55.9%)	49 (71.0%)	χ² = 2.32	0.128
Good sleep quality (PSQI ≤5)	15 (44.1%)	20 (29.0%)		

Independent samples t-tests were employed for continuous variables, while the chi-square test assessed the categorical association between gender and sleep quality. Although females had higher mean PSQI scores, the difference did not reach statistical significance in univariate analysis (p = 0.085). However, multivariable logistic regression (Table 5) identified female gender as a significant independent predictor of poor sleep quality [odds ratio (OR) = 2.43, 95% CI: 1.06-5.57, p = 0.04], after adjusting for migraine frequency and duration, indicating that gender exerts an independent predictive effect beyond the influence of migraine burden. Table 5 also demonstrates that higher migraine frequency was significantly associated with poor sleep quality (OR = 1.46 per additional attack/month, 95% CI: 1.18-1.82, p<0.001), whereas the duration of migraine history showed a non-significant trend (p = 0.11). These findings highlight the multifactorial nature of sleep disturbances in migraineurs, with both biological sex and headache burden playing critical roles. After adjusting for gender, age, migraine frequency and duration, depression severity (PHQ-9 score), and medication use, female gender (OR = 2.43, 95% CI: 1.06-5.57, p = 0.04) and migraine frequency (OR = 1.46 per attack/month, 95% CI: 1.18-1.82, p<0.001) remained significant independent predictors of poor sleep quality.

**Table 5:** Gender differences and logistic regression predictors of poor sleep quality (PSQI >5) ^*^P-value <0.05 indicates statistical significance CI: confidence interval; OR: odds ratio; PSQI: Pittsburgh Sleep Quality Index

Predictor	B (SE)	Wald	OR (95% CI)	P-value^*^
Gender	0.89 (0.43)	4.23	2.43 (1.06–5.57)	0.04
Migraine frequency (per attack)	0.38 (0.11)	11.91	1.46 (1.18–1.82)	<0.001
Duration of migraine (years)	0.11 (0.07)	2.52	1.11 (0.98–1.26)	0.11

## Discussion

This study examined the correlation between migraine frequency and sleep disturbances in adults, revealing a clear and statistically significant relationship. Our findings demonstrated that higher migraine frequency is associated with poorer sleep quality, as evidenced by increasing PSQI scores and a rising prevalence of sleep disturbances in participants with more frequent migraine attacks. Additionally, migraine intensity and duration were positively correlated with sleep disruption, while, in terms of gender differences, females experienced both more frequent migraines and poorer sleep quality. These results underscore the importance of evaluating sleep quality as part of comprehensive migraine management and suggest that interventions targeting sleep improvement could help reduce the overall migraine burden. Moreover, the study highlights the need for personalized treatment approaches that consider gender-specific factors influencing both migraines and sleep.

These results align closely with existing literature emphasizing the bidirectional relationship between migraines and sleep disturbances [20]. Increased migraine frequency has been linked to greater sleep problems, including difficulty falling asleep, fragmented sleep, and non-restorative sleep [21]. The strong positive correlation between migraine frequency and poor sleep quality suggests that migraines may exacerbate sleep disturbances and vice versa, creating a self-perpetuating cycle that adversely affects patient quality of life [22]. While our study focuses on the impact of migraine on sleep, we acknowledge the possibility of reverse causation, whereby poor sleep may also increase migraine frequency, a relationship that warrants further longitudinal investigation.

The observed gender disparity, with females reporting more frequent migraines and worse sleep quality, mirrors epidemiological data indicating that migraine prevalence and severity tend to be higher in women. This could be partially attributed to biological mechanisms such as fluctuations in estrogen levels, which are known to influence both migraine susceptibility and sleep regulation. Additionally, heightened stress reactivity and psychosocial stressors may further contribute to sleep disturbances in female migraineurs. Our findings on migraine intensity and duration also support previous research showing that more severe or chronic migraine conditions are commonly accompanied by greater sleep impairment [23]. These insights suggest that healthcare providers should be particularly attentive to sleep issues in women with migraine and consider tailored interventions that address hormonal and psychological contributors.

Compared to prior studies assessing sleep quality in migraine patients, our mean PSQI scores and prevalence of poor sleep quality fall within comparable ranges, supporting the external validity of our findings [24]. The significant association between migraine frequency categories and poor sleep prevalence further reinforces the idea that migraine burden directly impacts sleep health [25]. These results support the need for treatment plans that consider both migraine care and improving sleep quality using both behavioral and medication approaches. Ultimately, this study adds to the growing body of evidence advocating for a holistic, gender-sensitive approach in managing migraine patients: one that incorporates sleep hygiene, stress management, and broader lifestyle interventions alongside conventional headache therapies.

Limitations and future directions

This study has several limitations. Due to the cross-sectional design, causal relationships between migraine frequency and sleep disturbances cannot be definitively established. The reliance on self-reported data introduces the potential for recall and reporting bias. Although depressive symptoms were screened using the PHQ-9 and clinically significant psychiatric disorders were excluded via structured interviews, anxiety was not assessed with a standardized instrument such as the GAD-7, which may limit the assessment of its impact on sleep. Polysomnography was not conducted; however, patients with suspected obstructive or central sleep apnea were excluded based on clinical evaluation. Other potentially influential factors (such as specific medication use, lifestyle behaviors, and dietary patterns) were not systematically controlled for.

Future research should incorporate longitudinal designs to better determine the directionality of the migraine-sleep relationship. The inclusion of objective sleep assessments (e.g., polysomnography or actigraphy) alongside validated psychological screening tools would enhance the diagnostic accuracy. Moreover, interventional trials assessing the impact of targeted sleep-improvement strategies on migraine frequency and severity are warranted. Finally, exploring gender-specific biological and psychosocial mechanisms may help develop more personalized approaches to migraine management.

## Conclusions

This study revealed a significant positive association between migraine frequency and sleep disturbances, with greater migraine frequency linked to poorer sleep quality. These findings emphasize the importance of evaluating and managing sleep issues as a core component of migraine treatment, particularly among female patients who appear to be more affected. Improving sleep health may substantially reduce migraine burden and enhance overall quality of life. Therefore, routine assessment of sleep quality should be integrated into clinical care protocols for migraine sufferers. While our results suggest that enhancing sleep may help alleviate migraines, this remains speculative and requires confirmation through longitudinal and interventional studies. Reverse causality is also plausible: poor sleep may contribute to increased migraine frequency. The observed gender differences may reflect underlying biological mechanisms, including hormonal fluctuations and heightened stress reactivity in females, which merit further investigation.
